# Factors determining presence of passerines breeding within White Stork *Ciconia ciconia* nests

**DOI:** 10.1007/s00114-017-1492-2

**Published:** 2017-08-18

**Authors:** Adam Zbyryt, Dariusz Jakubas, Marcin Tobolka

**Affiliations:** 1 The Polish Society for Birds Protection (PTOP), Ciepła 17, 15-471 Białystok, Poland; 2 Department of Vertebrate Ecology and Zoology, University of Gdańsk, Wita Stwosza 59, 80-308 Gdańsk, Poland; 3 Institute of Zoology, Poznań University of Life Sciences, Wojska Polskiego 71C, 60-625 Poznań, Poland

**Keywords:** Nest, Breeding ecology, Nesting site selection, Communal breeding, Predator protection

## Abstract

Nests of White Stork *Ciconia ciconia* are commonly used by various passerines as nesting sites. In this study, we investigated factors determining presence and number of pairs of species breeding within White Stork nests in an extensive farmland in NE Poland. In 133 (57%) out of 233 White Stork nests, we found at least one breeding pair of passerine bird. These were from three species: House Sparrows *Passer domesticus* (68% of 133 nests with co-breeding), Tree Sparrows *Passer montanus* (65%), and Starlings *Sturnus vulgaris* (30%). The probability of breeding passerines within White Stork nests increased with increasing nest thickness, and was significantly higher in currently occupied nests. Sparrows were more likely to breed in White Stork nests located on electricity poles, situated closer to settlements and surrounded mainly by arable fields where meadows were not prevalent. In this paper, we show that White Stork nests are favorable nesting sites for passerines, as they are well insulated and provide an anti-predatory shield.

## Introduction

Many bird species are known to nest in assemblages of several pairs of the same or different species. Over 10% of avian species including gulls *Laridae*, auks *Alcidae*, waders *Charadrii*, some raptors *Accipitriformes*, and many passerines *Passeriformes* breed colonially (Gill 2007). This has several advantages such as increased predator detection (e.g., Elgar 1989), communal defense (e.g., Stenhouse et al. 2005; Jungwirth et al. 2015), and facilitated foraging (e.g., Møller 1987; Richner and Heeb 1995). However, it brings several costs due to stress and aggressive behavior (e.g., Saino 1994; Nicol et al. 1999) or parasite transfer (e.g., Valera et al. 2003).

One form of communal breeding is when smaller birds, for example, passerines, breed in the nests of bigger species. According to the “predator protection” hypothesis (Koskimies 1957), using the nests of other, bigger species may provide passerines with better protection from predators compared to individual breeding sites. Co-breeding species nesting near protective associates gain reproductive success benefits. More aggressive species or individuals are often chosen as a neighbor (Quinn and Ueta 2008). However, breeding in predators’ nests may be disadvantageous for small passerines. Some opportunistic predators prey on passerine chicks (Jaksić and Braker 1983). A particular case of colonial nesting is the co-breeding of passerines in White Stork (WS) *Ciconia ciconia* nests. In Europe, House Sparrow *Passer domesticus*, Tree Sparrow *P. montanus*, and Starling *Sturnus vulgaris* are the most common WS co-breeders (Indykiewicz 2006). The WS nest provides good shelter from precipitation, low temperatures, and wind for passerines and is available during the breeding (Indykiewicz 1998, 2006; Bocheński 2005; Kosicki et al. 2007) and wintering seasons (Tobolka 2007, 2011). On the other hand, the exposed location of the WS nests (on electricity poles, high chimneys, and the tops of buildings) increases predation risk (e.g., by the Sparrowhawk *Accipiter nisus*) during flight to/from the nest when it is used as a roosting site (Tobolka 2007, 2011). What is more, the WS may be a potential predator of residents as it is an opportunistic predator and it has been documented that passerine birds may be included in its diet (Kosicki et al. 2006; Chenchouni 2016). However, predation in the case of associated nesting occurs rarely. In Algeria, only a few cases of WS predation on sparrows within WS nests have been recorded (Chenchouni, personal information). Co-breeding with larger species may affect the behavior of the protected species. Savannah sparrows *Passerculus sandwichensis* nesting far from gull *Larus* spp. colonies were much more cautious while returning to their nests and perched on vegetation nearby for 20% longer before entering the nest compared to individuals breeding in gull colonies (Wheelwright et al. 1997).

Populations of Tree Sparrow and House Sparrow are currently declining in many European countries (Mitschke et al. 2000; Hole et al. 2002; Summers-Smith 2003, 2005; Robinson et al. 2005; Shaw et al. 2008), including Poland (Chylarecki and Jawińska 2007). Decreased nesting site availability, due to restoration of buildings and thermo-modernization limiting access to potential nesting sites, is considered as one of the most important factors causing population decline (Crick et al. 2002). In this context, WS nests additionally providing higher anti-predatory protection may be perceived as favorable breeding/roosting sites for passerines (Indykiewicz 1998, 2006; Tobolka 2011). So far, only a few papers have focused on the breeding of passerines within WS nests. Most of them are simply of a descriptive nature (e.g., Indykiewicz 1998, 2006; Bocheński 2005; Tobolka 2007, 2011) or investigate very simple and arbitrarily chosen factors determining the number of breeding pairs of Sparrows (e.g., Kosicki et al. 2007).

The aim of this study is to investigate factors determining presence/absence and number of species and pairs breeding within WS nests in an extensive farmland in NE Poland. We examine whether the presence/number of pairs of passerines breeding within WS nests is determined by WS nest thickness or nest location (electricity pole or other location). We hypothesize that thicker WS nests may accommodate more passerines. We expect that WS nests located on electricity poles attract more co-breeding passerines. One may expect that thicker nests may accommodate more co-breeding passerines. However, this hypothesis has not been confirmed (e.g., Kosicki et al. 2007; Tobolka 2011). Also, nest location may play an important role in passerine colonization of WS nests. Nests located on electricity poles are considered as safer from predators than to nests located on buildings or trees (Tryjanowski et al. 2009). Furthermore, we examine whether the number of breeding passerine pairs was affected by the main habitats in surrounding areas. Sparrows breeding close to human settlements usually forage in open agricultural areas like arable land, meadows, or set-asides (Shaw et al. 2008); therefore, we predict that the number of Sparrows and Starlings breeding in WS nests depends on the habitat structure in the vicinity of the nest. Due to possible inter-species competition between House and Tree Sparrows sharing the same foraging and nesting niches (Cordero and Rodriguez-Teijeiro 1990), we expect that those species will avoid co-nesting, which has been also shown in an urban environment (e.g., Węgrzynowicz 2012).

## Materials and methods

### Study area

The study was carried out in NE Poland, in the North Podlasie Lowland (21° 51′– 23° 57′ E; 52° 17′– 53° 54′ N) within four mesoregions: Biebrza Basin, Białystok Heights, Bielsk Plain, and Upper Narew Valley covering 9 440 km^2^ (Kondracki 2013). The postglacial landscape of North Podlasie Lowland is characterized by gently roughened relief, strongly transfigured by the denudation processes. These mesoregions are one of the least populated in Poland. The landscape is dominated by agricultural land which occupies ca. 60%, forests cover ca. 30%, while urban areas constitute less than 5%. The largest urban centre is Białystok (195,000 citizens). Farmland dominates the landscape of the studied area with arable lands making up ca. 60%, meadows ca. 30%, and pastures ca. 6% of total utilized agricultural area (Statistical Office 2016). This area is classified as a region dominated by intensive farming (Chylarecki et al. 2006) with dominance of small farms (up to 10 ha) with extensive agriculture use.

### Fieldwork

To collect data on the presence and numbers of co-breeding bird species, we surveyed in total 133 and 90 nests of WS occupied and unoccupied by passerines, respectively (in that 80 and 38 in 2014 and 53 and 52 in 2015, respectively). We studied different nests in both study years. The area selected for the study is characterized by the highest WS density in Poland (density of breeding pairs more than 40 pairs per 100 km^2^). The majority of WS nests in the North Podlasie Lowland are located on electricity poles (> 50%). In contrast to other parts of the country, the share of nests on roofs is still high constituting 30 % (Guziak and Jakubiec 2006).

We collected data in the first and second weeks of May, i.e., during the period of intense feeding of the young. We surveyed all nests only once. During the survey, we spent ca. 20 min (range 20–50 min) at each WS nest collecting data on nest status (occupied/not occupied by WS) and co-breeding species status (presence/absence, species, and number of pairs).

For each surveyed WS nest, we also collected the following set of variables: main type of agriculture within the radius of 500 m around the nest (classified as pasture, meadow, or arable land when that habitat covered > 75% of area), type of the nest location (electricity pole, roof, or tree), distance to the nearest building, nest thickness (the height of the nest construction), and nest occupation status (occupied or not by WS). Nest thickness was estimated with 10 cm accuracy by visual comparison with the width of the platform under WS nest (120 cm), the bricks of the chimney (width of 25 cm), and other electricity pole characteristics.

### Statistical analyses

To analyze factors determining presence/absence of avian species breeding within WS nests in NE Poland, we used logistic regression. To analyze factors affecting number of species and pairs breeding in WS nests, we used multiple ordinary least square (OLS) regression. Predictors used in all analyses are described in Table 1. Firstly, we analyzed presence/absence of any co-breeding passerine species. Then, we performed analyses only for WS nests with other avian species breeding within, separately for Starling, House Sparrow, and Tree Sparrow. For those uni-species models, we performed analyses in two steps: firstly, with a predictor describing presence/absence of any other co-breeding passerines (i.e., SAlien for Starling, HSAlien for House Sparrow, and TSAlien for Tree Sparrow) to recognize if the effect of any co-breeding species is important for the focal species. If this predictor was important, we performed an additional analysis with separate predictors indicating presence/absence of the particular co-breeding species (i.e., SPres, HSPres, TSPres) to recognize if the presence of a particular species is important for the focal species.

**Table 1:** Variables used in logistic and OLS multiple regression analyses (LR and OLSR, respectively)

Code	Variable	Comments	Analysis
AvPres	Presence of any avian species breeding in WS nest	0/1—absence/presence	LR
Arable	Prevalence of arable land within the radius of 500 m of WS nest	0/1—false/true	LR, OLSR
Mead	Prevalence of meadows within the radius of 500 m of WS nest	0/1—false/true	LR, OLSR
Pole	Nest situation on the pole	0/1—false/true	OLSR
WSPres	WS nest status	0/1—unoccupied/occupied	OLSR, LR
DisClosWS	Distance to the nearest WS nest	In meters	LR, OLSR
DisBuild	Distance to the nearest building	In meters	LR, OLSR
NestThick	Nest thickness	Expressed by its height (in centimeters)	LR, OLSR
NoSpec	No. of species breeding in WS nest	–	OLSR
TotNoPair	Total no. of pairs of breeding in WS nest	All species combined	OLSR
SPair	No. of Starling pairs breeding in WS nest	–	OLSR
HSPair	No. of House Sparrow pairs breeding in WS nest	–	OLSR
TSPair	No. of Tree Sparrow pairs in WS nest	–	OLSR
SPres	Presence of Starling breeding in WS nest	0/1—absence/presence	LR
HSPres	Presence of House Sparrow breeding in WS nest	0/1—absence/presence	LR
TSPres	Presence of Tree Sparrow breeding in WS nest	0/1—absence/presence	LR
SAlien	Presence of species other than Starling in WS nest	0/1—absence/presence of HS and TS combined	OLSR
HSAlien	Presence of species other than House Sparrow in WS nest	0/1—absence/presence of S and TS combined	OLSR
TSAlien	Presence of species other than Tree Sparrow in WS nest	0/1—absence/presence of S and HS combined	OLSR

*WS* White Stork

We assessed whether the data sufficiently met the assumptions of the linear model using Q-Q plots (quantile expected in normal distribution vs quantile observed for residuals). We normalized data on DisClosWS, DisBuild, and NestThick using the ln transformations. All analyzed predictors were non-collinear (Pearson correlation coefficients, *r* < |0.15|).

We selected the best logistic and OLS regression models using Akaike’s information criterion for small sample sizes (AIC_c_) (Burnham and Anderson 2002; Hegyi and Garamszegi 2011). We compared the relative performance of the models based on ∆AIC_c_, i.e., difference between the AIC value of the best model and the AIC value for each of the other models (Burnham and Anderson 2002). We presented only models with ∆AICc ≤ 1, suggested to be within the range of plausible models to best fit the observed data (Burnham and Anderson 2002).

The significance of the OLS regression models was checked using Wald statistics. To evaluate the discriminatory performance of logistic regression models, we used the receiver operating characteristic function (AUC) (Pearce and Ferrier 2000). Our criteria for the predictive capability were based on the AUC value. We considered logistic regression models with AUC ≥ 0.7 as good models and < 0.7 as poor models (Hosmer et al. 2013). We presented only OLS regression models with at least one significant (*p* > 0.05) predictor.

We performed all statistical analysis in R software (R Development Core Team 2015) with MuMin (Bartoń 2013), aod (Lesnoff and Lancelot 2012), and pROC (Robin et al. 2011) packages.

## Results

### Frequency of nesting passerines within White Stork nests

In 133 White Stork nests (57% of all surveyed), we found at least one of three co-breeding passerine species, i.e., House Sparrows (68% of all nests with co-breeding), Tree Sparrows (65%), and Starlings (30%). Breeding frequency differed significantly between them (*χ* ^2^_2_ = 15.74, *p* < 0.001). The proportion of WS nests with House Sparrows was similar to that with Tree Sparrows (*χ* ^2^_1_ = 0.054, *p* = 0.82). Starlings nested in WS nests, less frequently than House Sparrows (*χ* ^2^_1_ = 13.5, *p* = 0.0002) or Tree Sparrows (*χ* ^2^_1_ = 11.9, *p* = 0.0005). We found nesting of more than one species in 51% of the WS nests with co-breeding passerines. Some WS nests were occupied exclusively by only one passerine species (33% of WS nests with Starlings, 32% of nests with House Sparrows, and 49% of nests with Tree Sparrows).

### Factors determining presence/absence of avian species breeding within White Storks nests

For all passerine species combined, we found two logistic regression models describing the factors affecting the presence/absence of co-breeding within WS nests (Table 2). In both models, the probability of breeding passerines within WS nests increased with increasing nest thickness, and was significantly higher in nests currently occupied by WS, situated on electricity poles, and characterized by low prevalence of meadows in the vicinity. Additionally, according to the second candidate model [with higher predictive capability (AUC = 0.78) compared to the first one (AUC = 0.71)], presence of co-breeders was more likely in nests situated closer to buildings (Table 2).

**Table 2:** Rank of the best logistic regression models for factors determining presence/absence of species breeding within White Storks nests in NE Poland based on Akaike’s information criterion corrected for small sample size (AIC_c_)

Model type	Model parameters	AIC_c_	∆AIC_c_	Akaike’s weights	AUC
FM	AvPres **~** arab + mead + pole + WSPres + DisBuild + NestThick + DisClosWS				
BMs	−Int + NestThick − mead − DisBuild + pole + WSPres	210.5	0.00	0.373	**0.71**
	−Int + NestThick − mead + pole + WSPres	211.7	1.20	0.205	**0.78**
FM	SPres ~ arab + mead + pole + WSPres + DisBuild + NestThick + NestSit + DisClosWS + HSPres + TSPres				
BMs	−Int + NestThick	164.6	0.00	0.239	0.57
	−Int	164.7	0.13	0.224	−
FM	HSPres ~ arab + mead + pole + WSPres + DisBuild + NestThick + NestSit + DisClosWS + SPres + TSPres				
BMs	Int − mead − BuildDis − TSPres + pole	155.0	0.00	0.220	**0.72**
	Int + arab − mead − BuildDis − TSPres + pole	155.0	0.02	0.217	**0.70**
	Int + arab − BuildDis − TSPres + pole	155.4	0.44	0.176	0.68
	Int − mead − BuildDis − TSPres + pole − SPres	155.9	0.89	0.141	**0.74**
FM	TSPres ~ arab + mead + pole + WSPres + DisBuild + NestThick + NestSit + DisClosWS + HSPres + SPres				
BMs	Int + mead − HSPres	165.6	0.00	0.239	0.44
	Int − HSPres	166.2	0.64	0.174	0.37
	Int + mead − HSPres + pole	166.3	0.68	0.170	0.48
	Int + mead − HSPres + DisBuild	166.6	0.99	0.146	0.55

Only the best models with ∆AIC_c_ ≤ 1 are presented; Akaike’s weights are calculated from the full set of models. The predictive capability of functions based on area under the receiver operating characteristic function (AUC); good models (AUC ≥ 0.7) bolded. Predictor codes: see Table 1

*FM* full model, *BMs* best models, *Int* intercept

Then, we performed separate analyses for particular co-breeding species. For the Starling, we found two models describing the factors affecting breeding within WS nests. According to the first model, the probability of presence of Starlings increased with increasing nest thickness. However, its predictive capability was low (AUC = 0.57). The second model includes only an intercept (Table 2).

For the House Sparrow, we found four models describing the factors affecting their breeding within WS nests (Table 2). In the fourth model [with the highest predictive capability (AUC = 0.74)], the probability of presence of House Sparrows was higher in WS nests situated in areas where meadows are not prevalent (92% of nests), closer to buildings, without breeding Tree Sparrows, without Starlings (however, this effect was not significant, *p* = 0.25), and situated on electricity poles (68% of nests) (Table 2).

For the Tree Sparrow, we found four models describing factors affecting their breeding within WS nests (Table 2). According to the model with highest predictive capability (AUC = 0.55), the probability of presence of Tree Sparrows was higher in WS nests without co-breeding House Sparrows, and situated in areas with high prevalence of meadows, located further from buildings. However, prevalence of meadows and distance from buildings were not significant (*p =* 0.139, *p =* 0.288) (Table 3).

**Table 3:** The best logistic regression models for factors determining presence/absence of species breeding within White Storks nests in NE Poland

Model parameters	*b*	SE	*P*
All species combined (model 2, AUC = 0.779)			
Int	−9.09	1.93	<0.001
NestThick	1.73	0.47	<0.001
Mead	−1.00	0.46	0.030
Pole	2.90	0.42	<0.001
WSPres	1.69	0.45	<0.001
Starling (model1, AUC = 0.75)			
Int	−3.61	1.90	0.057
NestThick	0.73	0.50	0.141
House Sparrow (model 4; AUC = 0.742)			
Int	1.26	0.77	0.104
Mead	−1.15	0.60	0.056
DisBuild	−0.47	0.16	0.004
TSPres	−1.24	0.50	0.013
Pole	1.95	0.96	0.043
SPres	−0.51	0.45	0.253
Tree Sparrow (model 4; AUC = 0.55)			
Int	1.13	0.50	0.025
Mead	1.18	0.80	0.139
DisBuild	0.12	0.11	0.288
HSPres	−1.14	0.48	0.017

Predictor codes: see Table 1

*Int* intercept

### Factors affecting numbers of species and pairs breeding within White Stork nests

All OLS regression models investigating factors affecting the number of species breeding within WS nests were characterized by very low determination coefficients (adjusted *R* ^2^ < 0.01). According to the best OLS regression model determining factors affecting the total number of co-breeding pairs (adjusted *R* ^2^ = 0.11), their number was higher in areas surrounded by arable land, in thicker nests, nests situated closer to buildings, and nests on electricity poles (Table 4).

**Table 4:** Rank of the best OLS regression models for factors affecting number of species and pairs breeding within White Storks nests in NE Poland based on Akaike’s information criterion corrected for small sample size (AIC_c_)

Model type	Model parameters	AIC_c_	∆AIC_c_	Akaike’s weights	Wald test (*p*)	Adj. *R* ^2^
FM	NoSpec ~ arab + mead + pole + WSPres + DisBuild + NestThick + DisClosWS					
BMs	Int	283.1	0	0.266	< 0.001	−
	Int − DisBuild + pole	283.9	0.86	0.173	< 0.001	0.01
	Int + pole	284	0.93	0.167	< 0.001	0.001
FM	TotNoPair ~ arab + mead + pole + WSPres + DisBuild + NestThick + DisClosWS					
BMs	−Int + arab + NestThick − DisBuild + pole	524.6	0	0.378	< 0.001	**0.107**
FM	SPair1 ~ arab + mead + pole + WSPres + DisBuild + NestThick + NestSit + DisClosWS + SAlien					
BMs	−Int + NestThick − SAlien	272.3	0	0.238	< 0.001	0.099
	−Int + NestThick + DisClosWS − SAlien	272.5	0.24	0.211	< 0.001	**0.105**
	−Int + NestThick − mead + DisClosWS − SAlien	273	0.73	0.165	< 0.001	**0.11**
FM	SPair2 ~ arab + mead + pole + WSPres + DisBuild + NestThick + NestSit + DisClosWS + HSPres + TSPres					
BMs	−Int + NestThick	280.9	0	0.237	< 0.001	0.031
	−Int + NestThick + pole	281.6	0.68	0.169	< 0.001	0.034
FM	HSPair1 ~ arab + mead + pole + WSPres + DisBuild + NestThick + NestSit + DisClosWS + HSAlien					
BMs	Int + arab − mead − DisBuild − HSAlien + pole	437.1	0	0.428	< 0.001	**0.133**
FM	HSPair2 ~ arab + mead + pole + WSPres + DisBuild + NestThick + NestSit + DisClosWS + SPres + TSPres					
BMs	Int + arab − mead − DisBuild − TSPres + pole	434.8	0	0.252	< 0.001	**0.148**
	Int + arab − DisBuild − TSPres + pole	435.4	0.58	0.188	< 0.001	**0.137**
FM	TSPair1 ~ arab + mead + pole + WSPres + DisBuild + NestThick + NestSit + DisClosWS + TSAlien					
BMs	−Int + NestThick + arab + mead − TSAlien + pole	409.4	0	0.205	< 0.001	0.081
	−Int + NestThick + arab + mead − TSAlien	409.5	0.09	0.196	< 0.001	0.088
FM	TSPair2 ~ arab + mead + pole + WSPres + DisBuild + NestThick + NestSit + DisClosWS + SPres + TSPres					
BMs	−Int + arab + mead + NestThick	409.5	0	0.24	< 0.001	0.072
	−Int + arab + mead + NestThick + pole	409.7	0.11	0.228	< 0.001	0.079
	−Int + arab + mead + NestThick − HSPres	410.4	0.85	0.157	< 0.001	0.074

Only the best models with ∆AIC_c_ ≤ 1 are presented; Akaike’s weights are calculated from the full set of models. Adj *R* ^2^—adjusted coefficient of determination (values > 0.10 bolded). Predictor codes: see Table 1

*FM* full model, *BMs* best models, *Int* intercept

Then, we performed separate models for particular co-breeding species. For the Starling, we found three models describing the factors affecting the number of pairs breeding within WS nests (Table 4). According to the best model (with the highest determination coefficient, adj. *R* ^2^ = 0.11), the number of breeding pairs was higher in thicker WS nests without any co-breeding passerines, situated further from the nearest WS nest, and where meadows are not prevalent. However, distance to nearest WS nest and prevalence of meadows were not insignificant (*p* > 0.08) (Table 5). When considering the presence/absence of particular co-breeding species, we found two models. In both models, the number of Starling pairs was higher in thicker WS nests (*p* = 0.02). According to the second model, number of pairs was also higher in nests situated on electricity poles; however, this effect was not significant (*p* = 0.23) (Table 5). Both models were characterized by low determination coefficients (adj. *R* ^2^ < 0.04).

**Table 5:** The best OLS regression models for factors affecting number of species and pairs breeding within White Storks nests in NE Poland

Model parameters	*B*	SE	*p*
All pairs combined (TotNoPair, model1, adj. *R* ^2^ = 0.107)			
Int	−1.81	1.56	0.249
Arab	1.44	0.56	0.011
Pole	1.74	0.65	0.008
DisBuild	−0.25	0.11	0.031
NestThick	0.91	0.39	0.020
Starling1 (SPair, model3, adj. *R* ^2^ = 0.110)			
Int	−0.76	0.66	0.258
Mead	−0.24	0.19	0.200
NestThick	0.40	0.15	0.009
SAlien	−0.86	0.26	0.001
DisClosWS	0.10	0.06	0.090
House Sparrow1 (HSPair, model1, adj. *R* ^2^ = 0.133)			
Int	1.33	0.38	0.001
Habit	0.87	0.40	0.032
DisBuild	−0.64	0.33	0.052
HSAlien	−0.19	0.08	0.021
Pole	−0.69	0.26	0.008
House Sparrow2 (HSPair, model 1, adj. *R* ^2^ = 0.148)			
Int	1.28	0.36	0.001
Arab	0.91	0.40	0.025
Past	−0.54	0.33	0.102
DisBuild	−0.19	0.08	0.022
Pole	0.92	0.46	0.049
TSPres	−0.69	0.22	0.003

Predictor codes: see Table 1

*Int* intercept

For the House Sparrow, we found one model describing the factors affecting the number of pairs breeding within WS nests (adj. *R* ^2^ = 0.13) (Table 4). The number of breeding pairs was higher in WS nests surrounded mainly by arable land (mean ± SD 2.2 ± 1.2 pairs vs 1.2 ± 1.3 pairs), where meadows are not prevalent (1.4 ± 1.3 pairs vs 0.6 ± 0.8 pairs), in WS nests situated closer to buildings, without co-breeding passerines (1.8 ± 1.1 pairs vs 1.3 ± 1.3 pairs), and situated on electricity poles (1.3 ± 1.3 pairs vs 1.1 ± 1.2 pairs) (Table 5). In the set of models including presence/absence of particular co-breeding species, the best model (adj. *R* ^2^ = 0.15) predicts that the probability of breeding House Sparrows is higher in WS nests surrounded mainly by arable land, where pastures are not prevalent, situated closer to buildings, without co-breeding Tree Sparrows, and situated on electricity poles (Table 5).

For the Tree Sparrow, we found two models describing the factors affecting the number of pairs breeding within WS nests (Table 4). In both models, the number of breeding pairs was higher in thicker WS nests, in areas surrounded by arable land (mean ± SD 1.8 ± 1.5 pairs vs 1.0 ± 1.1 pairs in areas surrounded mainly by other habitats) and meadows (1.6 ± 1.3 pairs vs 1.0 ± 1.1 pairs), and without co-breeding passerines (1.5 ± 0.9 pairs vs 1.0 ± 1.2 pairs). According to the first model, the number of pairs was also higher in nests situated on electricity poles (Table 5). However, both models were characterized by very low determination coefficients (adj. *R* ^2^ < 0.09). When considering the presence/absence of particular co-breeding species, we found three models. In all models, the number of breeding pairs was higher in thicker WS nests, located in areas surrounded by arable land and meadows. According to the second model, the number of pairs was also higher in nests situated on electricity poles, and according to the third model, in nests without co-breeding House Sparrows (Table 5). However, all models were characterized by negligible determination coefficients (adj. *R* ^2^ < 0.08).

## Discussion

This is the first study investigating factors affecting breeding of passerines within WS nests in NE Poland, i.e., in the optimal WS breeding area in Central Europe (Profus 2006), characterized by high breeding density (Guziak and Jakubiec 2006). So far, studies on co-breeding passerines were conducted in areas with low or very low WS density in W Poland (e.g., Indykiewicz 2006; Kosicki et al. 2007; Tobolka 2007, 2011).

### Factors determining presence and numbers of co-breeding passerines within White Stork nest

We found that over 50% of the studied WS nests had co-breeding passerines. Breeding within WS nests has several advantages such as good thermal insulation (Pinowski et al. 2006) and anti-predator protection (Indykiewicz 2006). Our analyses revealed that the probability of breeding of any passerines within WS nests increased with increasing nest thickness and distance from the nearest building, and was higher in currently occupied nests and in areas where meadows are not prevalent. Our analyses indicate that nest occupancy by WS was an important factor, suggesting that its presence may be advantageous for co-breeding passerines. This is in concordance with other authors’ suggestions that presence of WS serves as an anti-predator shield for co-breeding passerines (e.g., Indykiewicz 2006). It may be also explained in terms of differing construction structure of occupied vs unoccupied nests. Nests regularly occupied by WS pairs have more space between twigs, and there are many niches in the upper part enabling nest building by co-breeding passerines. In contrast, WS nests unoccupied for several years have no niches and are more compressed, which constrains penetration by passerines. However, it has been shown that even abandoned WS nests may still constitute attractive nesting site for one to two pairs of Tree Sparrow and House Sparrow (Boratyn 2015).

Among models investigating both presence and number of pairs of a particular species, the House Sparrow models were characterized by the highest predictive capability. For both presence and number of pairs, habitat type, distance to the nearest building, and presence of co-breeding passerines were recognized as important factors. House Sparrows preferred to breed within WS nests situated on electricity poles, surrounded mainly by arable land, situated closer to buildings, and without co-breeding passerines, especially the Tree Sparrow. Building nests on electricity poles has several advantages both for the nest owner and for the co-breeding species. Such locations increase protection against mammalian predators, e.g., Martens *Martes* sp. This advantage has been postulated as the main reason of changes in nest locations in WS during the last 40 years in W Poland (Tryjanowski et al. 2009). However, such exposed locations may increase the risk of being predated (e.g., Tobolka 2007) and do not protect the nest owners’ nestlings (and probably also co-breeding species) against severe weather conditions (e.g., Moreno and Møller 2011; Janiszewski et al. 2015; Tobolka et al. 2015).

We recognized prevalence of arable lands in close proximity of WS nests as another important predictor of House Sparrow breeding. Preference for nesting in WS nests located in areas with high prevalence of arable land was surprising because often farmland bird species abundance decreases with increasing area of arable land (Donald et al. 2001). However, the direction of this relationship may depend on the availability of that habitat at the regional scale—for example, some regions of Britain scarce in arable habitat are characterized by increased bird numbers (Robinson et al. 2001). In our study area, arable land is less common than meadows, pastures, or forests. In traditionally cultivated, organic agricultural farmland of NE Poland, with a low rate of herbicide use (Adamczewski and Dobrzański 2012), arable land serves as attractive foraging habitat for Sparrows. Meadow and pastures are less attractive, because vegetation there is usually lower, and plants are not allowed to produce fruits due to regular grazing or mowing. Although Sparrows need invertebrates during the breeding season for feeding young nestlings, they need plant seeds to feed themselves and older nestlings (Anderson 2006). Farms provide other sources of food: in more traditionally managed farms, which dominate in our study area, animals are kept outside the buildings, hence the food for them is also available for birds, in this case for Sparrows. In WS nests located in or in close proximity to traditional farms, the number of nesting House Sparrows was higher compared to those breeding close to modern farms (Kosicki et al. 2007). The significant preference of House Sparrows to locate their nests in WS nests on electricity poles and closer to buildings has been also reported from north-central Poland (Indykiewicz 2006).

### Inter-specific competition

Finally, we found that the House Sparrow was more often recorded in WS nests without breeding Tree Sparrows. This relationship may result from high inter-species competition. Firstly, the House Sparrow is a little bigger than the Tree Sparrow (Cramp 1998) and known for aggressive behavior and high territoriality during breeding (e.g., Gowaty 1984; Wingfield et al. 1987), which has not been reported for Tree Sparrow (Cordero and Senar 1990). Secondly, the House Sparrow is a very flexible nester, nesting in many different locations including apertures and holes in buildings but also on antennas or bushes (Cramp 1998). Therefore, it can occupy more sites in WS nests and prevent nesting of other species. House and Tree Sparrows have similar habitat preferences; therefore, they compete for food and nest sites in areas of sympatric occurrence, especially in areas with domination of the latter species (Cordero and Rodriguez-Teijeiro 1990; Summers-Smith 1995). This may explain avoidance of co-breeding by both sparrow species in WS nests revealed in our study. The number of breeding Starlings was also higher in WS nests without presence of other passerines. This might also be explained in terms of inter-species competition or different nest site preferences.

### Other potential factors

Low predictive capabilities of many of the presented logistic regression models and low determination coefficients in OLS regression models suggest that there are more unstudied factors affecting the number of pairs of particular species breeding within WS nests. For the House Sparrow, it may be the number of predators in the area. In Poland, free-ranging domestic cats *Felis domesticus* hunt ca. one bird per cat per month, mostly Sparrow, and the real number of predated birds is predicted to be much higher. The highest predation occurs in June when juvenile Sparrows leave the nests (Baker et al. 2005; Krauze-Gryz et al. 2016). In British villages, the domestic cat is a major House Sparrow predator responsible for up to 30% of its mortality (Churcher and Lawton 1987). Hence, the number of cats in the area may affect the decision about breeding within WS nests. The number of another predator, the Sparrowhawk, may also serve as an important factor affecting the decision on breeding within WS nests. The population of this avian raptor increased 13-fold during the last 30 years in the study area (Pugacewicz 2010). Summers-Smith (2005) suggested that increasing Sparrowhawk predation, added to that from domestic and feral cats, may pose a serious threat to the House Sparrow population. Moreover, the availability of other potential nesting sites (buildings, hollow trees, haystacks, etc.) may be a significant factor determining sparrow presence in WS nest (e.g., Indykiewicz 1991).

## Conclusions

Our study revealed that > 50% of studied WS nests in NE Poland contained co-breeding passerines. We recognized nest location, distance to the nearest building, arable land prevalence, and presence of other co-breeding passerines as the main determinants of passerine nesting within WS nests in NE Poland. Breeding in WS nests is favorable for passerines as they provide an anti-predatory shield and a well-insulated nesting site. Further studies comparing breeding success in nests within or outside a WS nest are needed to fully comprehend the advantages and disadvantages of breeding in WS nests.

We are aware that our limited dataset (based on a restricted number of predictors) is insufficient for a comprehensive investigation of factors affecting breeding of passerines in WS nests. The data presented here can, however, be treated as a pilot study contributing to the planning of a broad-scaled investigation including local populations of passerine species that may nest in White Stork nests. The question of intra-species competition for nesting within WS nests should be addressed in future studies.
